# Epidemiology of Dog Bite Incidents in Chile: Factors Related to the Patterns of Human-Dog Relationship

**DOI:** 10.3390/ani11010096

**Published:** 2021-01-06

**Authors:** Carmen Luz Barrios, Carlos Bustos-López, Carlos Pavletic, Alonso Parra, Macarena Vidal, Jonathan Bowen, Jaume Fatjó

**Affiliations:** 1 Cátedra Fundación Affinity Animales y Salud, Universitat Autónoma de Barcelona, Parque de Investigación Biomédica de Barcelona, C/Dr. Aiguader 88, 08003 Barcelona, Spain; jaumefatjo@gmail.com; 2 Escuela de Medicina Veterinaria, Facultad de Ciencias, Universidad Mayor, Camino La Pirámide 5750, Huechuraba, Región Metropolitana 8580745, Chile; macarena.vidal@umayor.cl; 3 Departamento de Ciencias Básicas, Facultad de Ciencias, Universidad Santo Tomás, Santiago, Chile, Av. Ejército Libertador 146, Santiago, Región Metropolitana 8320000, Chile; cbustoslz@yahoo.es; 4 Departamento de Zoonosis y Vectores, Ministerio de Salud, Enrique Mac Iver 541, Santiago, Región Metropolitana 8320064, Chile; cpavletic@minsal.cl (C.P.); alonsoparra@minsal.cl (A.P.); 5 Queen Mother Hospital for Small Animals, Royal Veterinary College, Hawkshead Lane, North Mymms, Hertfordshire AL9 7TA, UK; jbowen@rvc.ac.uk

**Keywords:** bites, dog bites, dog aggression, epidemiology

## Abstract

**Simple Summary:**

Dog bites are a major public health problem throughout the world. The main consequences for human health include physical and psychological injuries of varying proportions, secondary infections, sequelae, risk of transmission of zoonoses and surgery, among others, which entail costs for the health system and those affected. The objective of this study was to characterize epidemiologically the incidents of bites in Chile and the patterns of human-dog relationship involved. The results showed that the main victims were adults, men. The dogs most involved in these incidents were medium-sized, mixed-breed, and most of these were known to the victim. The greatest frequency of such episodes occurred inside the home. This characterization of the problem is essential for a comprehensive understanding of the topic to develop successful dog bite prevention and management programs.

**Abstract:**

Dog bites are one of the main public health problems. They produce important consequences for those who suffer them (physical and psychological injuries, secondary infections, sequelae, risk of transmission of zoonoses and surgeries, among others). The objective of this study was to characterize epidemiologically the incidents of bites in Chile and the patterns of human-dog relationship involved. The records analyzed in this article were obtained from bitten patients who attended the main public health facilities in Chile during the period 17 September 2017 and 17 September 2018: In the period studied, 17,299 animal bites were recorded; however, only 7220 (41.74%) cases were analyzed in which the offending species could be identified. Of the bites analyzed, 6533 were caused by dogs (90.48%). Of these, 41.05% were caused by medium-sized dogs. Most bites were caused by dogs of mixed breeds (55.99%), followed by dogs of the German Shepherd breed (8.50%). Most of the dogs that bit were known to the victim (99.95%) and most of the attacks occurred indoors (57.48%). Although dog bite records have improved in Chile, it would be useful to also include background information on the context in which the incident occurred, which would be very useful for developing effective bite prevention programs.

## 1. Introduction

Dog bites are a major public health problem worldwide [1,2,3]. As a result of these incidents, important consequences emerge, among which are physical injuries, psychological trauma, zoonotic disease transmission [4,5,6], infections [7,8,9], dysfunction of injured body parts and economic costs [10], both for the state of the country in question, as well as for the victims of these episodes. Internationally, in underdeveloped and developing countries, highly lethal zoonotic diseases such as rabies occur, which is mainly transmitted by free-roaming dog bites [9], estimating that 99% of infections are produced by this type of incident [11]. In Chile, endemic rabies virus variants are present that can affect both wild and domestic animals [11]. The cases that have been evidenced in this country have mainly affected the insectivorous bat *Tadarida brasiliensis* [12]. It should be noted that Chile has been declared free of canine rabies virus variants since 2010 [13,14]. To maintain this status, notification is mandatory in humans and animals, surveillance is maintained, and viral variant identification is made in 100% of cases identified by the national rabies control program [15].

It has often been considered that there are important differences between the incidence of canine bites in developed countries and underdeveloped or developing countries. The latter have inadequate conditions for keeping animals, specifically associated with low levels of restriction, allowing the animals to roam freely and in public spaces temporarily or permanently [16], estimating a higher incidence of bites in underdeveloped countries than in developed countries [17]. However, only relying on the number of animals present on public roads or the level of development of each country is insufficient to analyze the problem comprehensively, as the arguments focus mainly on economic parameters such as participation in conglomerates of more developed countries like the Organization for Economic Cooperation and Development (OECD) [18]. Likewise, if we use indicators focused on a person’s development such as the Human Development Index belonging to the United Nations (UN) [19], which assesses life expectancy, education and standard of living, we can have useful information to understand this problem in the different latitudes of the world; however, this will not be specific or sufficient. This is because the evaluation dimensions in these types of indicators do not consider the human-animal interaction, which can lead to confusion when making decisions to control or prevent the problem of bites. An example of this is the case of Chile, a country that despite being classified by the UN as a country with high human development like Norway, Switzerland and the United States, belonging to the select group of the OECD and having a human-dog ratio very similar to that of the United States (CH: 4.1:1; US: 4.3:1) [20,21], presents a reality of dog demographics very similar to that of underdeveloped or developing countries. This last statement is based on the high percentages of dogs with owners that roam the streets (27%, 50% and 67% in cities, towns and rural areas, respectively) [22] and dogs that have no known owner (74%, 51% and 21% in cities, towns and rural areas, respectively) [22]; in the high number of dog bites (91.6%) [23] and in that the majority of canine bites occurred in the street [24]. Some of these figures coincide with those found in Kenya, where 69% of owned dogs moved without restriction [25], the majority of bite incidents were by dogs (93%) [26] and the highest number of these incidents were caused by dogs that roamed the streets freely (78%) [26].

Therefore, although the level of development of a population can help us in the basic understanding of the problem, to be able to comprehensively analyze the problem of canine bite incidents, there must be an exhaustive epidemiological characterization of the variables associated to the victim, the aggressor animal and the attack context. In addition, this must be complemented with a deepening of the patterns of human-dog coexistence, which includes some characteristics such as the daily habits of the victim and the relationship to the attack of certain types of dogs (type of confinement), places of higher interaction between animals and people, time of interaction between victims and biting animals and characteristics of these activities, among others. It should also include site-specific factors such as location, industrialization and cultural factors [27].

Given the above, the objective of this study is to describe the epidemiological reality of canine bites in Chile, as well as to analyze the factors related to the patterns of human-dog coexistence that can influence the occurrence of these incidents.

## 2. Materials and Methods

### 2.1. Participants and Materials

The records analyzed in this article were obtained from bitten patients who attended the main public health facilities in Chile, male and female and of all ages, for the period between 17 September 2017 and 17 September 2018.The data were collected by health personnel at the time of care. The bitten patients were registered in a digital platform called “Biting Animals Registry System” (BARS) [28], which has been developed and maintained by the Chilean Ministry of Health for rabies surveillance and monitoring of bites in the national territory.

### 2.2. Methodology

At first, 17,299 records of victims and animals participating in bite incidents were collected, as well as characteristics of the context of the attack, recorded on the BARS platform, from 17 September 2017 to 17 September 2018 in Chile. Of these records, only 6533 belonged to canine bites, and the present research focused and deepened on these. These antecedents were initially analyzed in a general way, where dog bites were distributed by region of occurrence. Subsequently, they were classified into three groups: victim’s background, the background of the aggressor animal and information about the context and consequences of the attack. The first group considered the victim’s gender and age group. The second included the species of the biting animal, breed, size, reproductive status, the relationship of the affected person with the biting animal and possession situation (with or without owner and residence). Finally, in relation to the context of the attack, seasonality, location where the event occurred, type of bite, and initiation of rabies treatment were incorporated (Table 1). All this information was obtained from BARS and in some cases, as an age group, grouped for the convenience of the analysis carried out in this study.

### 2.3. Statistic Analysis

A descriptive analysis of the data obtained in the sampling was carried out, using frequency tables. Subsequently, a comparison was made between the different variables using the Chi-square test of homogeneity and comparison of proportions, with a confidence level of 95%. In the case of the first test, it was carried out to determine if a categorical data set follows a multinomial distribution with certain proportions and to evaluate whether two discrete variables in our study were associated, comparing them with each other. In the case of the analysis of proportions, it was carried out to infer about the difference between two proportions of the variables used in the present study, considering significant a value of Z = 1.96 and *p* = 0.05. These analyses were carried out using the statistical program Minitab^®^ v.16

## 3. Results

In the present study, 17,299 bite incidents registered in the “Biting Animals Registry System” (BARS) of the Ministry of Health of Chile were analyzed, which corresponded to 100% (17,299/17,299) of the patients treated for this cause in the main public health services for primary and emergency care, during the period 17 September 2017 to 17 September 2018 [28]. It should be noted that these records only considered incidents that required medical attention and, therefore, incidents of a minor nature were not included. This is because such incidents usually do not go as far as requesting attention, as it may not be necessary. It is important to consider that they have not been voluntarily eliminated by the researchers for this study. The use of the data used in this study was facilitated by the Ministry of Health of Chile, through the issuance of document ORD.B38/N° 2316. Likewise, patients have been de-identified by providing specific data for the variables of interest to those in charge of analysing the sample. The data was processed in accordance with the guidelines of the Law for the protection of privacy N° 19628 of the Government of Chile [31].

The study included 6533 dog bites, which corresponded to 37.77% (6533/17,299) of the total recorded incidents (Table 2).

When grouping the bite incidents according to the administrative division of the country, the regions that reported the highest number of dog bites were the Valparaíso Region and the Metropolitan Region (Table 3).

### 3.1. Victim’s Information

#### Victim’s Gender

In relation to victim’s gender, men (52.15%) (3406/6531) were significantly more bitten than women (47.85%) (3125/6531) (Chi-Square = 12.0902; *p* = 0.001) (Table 4). It is important to consider that 6531 records had this information, which corresponded to 99.96% of the data.

Regarding the victim’s age group, of the 6533 records of dog bites, the interval with the highest number of incidents was that of >40 to 64 years (Group 6, with 0.82 bites/10,000 inhabitants). This group was followed by Group 5 (>25 to 40 years), which contributed to a rate of 0.52 bites/10,000 inhabitants. Finally, the least affected was Group 3 (0.29 bites/10,000 inhabitants) (Table 5). It should be noted that 94.84% (6196/6533) of the canine bite records had this information and that there was a significant difference between age groups (Chi-Square = 703.099; *p* = 0.001).

When crossing the variables age group and victim’s gender, it was observed that the age group with the highest number of bites in male victims was Group 6 (>40–64 years), followed by Group 5 (>25–40 years), with significant differences between both (Z = 3.39; *p* = 0.001). For female, the highest percentage of records (13.49%) (836/2970) was also found in Group 6 (>40–64 years), followed by Group 5 (>25–40 years) (Z = −3.39; *p* = 0.001). However, in the latter case, there were no statistically significant differences. When comparing male and female, there were significant differences between almost all age groups, except in Group 4 (>14–25 years) (Chi-Square = 2.23; *p* = 0.136) and 5 (>25–40 years) (Chi-Square = 2.89; *p* = 0.089) (Table 5).

### 3.2. Biting Animal

#### 3.2.1. Biting Animal Species

Only 41.74% (7220/17,299) of the files had information about the biting animal species. Among the data actually recorded, the dog was the most common species, causing 90.48% (6533/7220) of the incidents, followed by the cat with 9.52% (687/7220) (Table 6).

In relation to the distribution of bites by region according to the biting animal species, it was found that the highest number of dog bites occurred in the Valparaíso Region (26.47%) (1911/7220), followed by the Metropolitan Region (26.44%) (1909/7220). There was an association between the biting animal species and the attack region (Chi-Square = 51.757; *p* = 0.001) (Table 7).

For dog bites, there were significant differences between all regions (Chi-Square = 12,781; *p* = 0.001). It is important to note that significant differences were found between the Metropolitan Region and the Valparaíso Region (Z = 2.82; *p* = 0.005).

#### 3.2.2. Size and Breed of the Biting Animal

**Figure 1:**
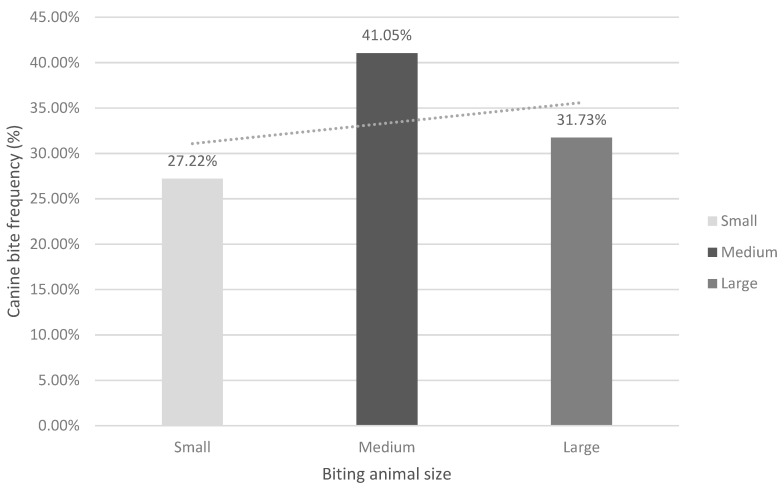
Distribution of canine bites according to the biting animal size.

The results associated with the size of the biting animal showed that medium-sized dogs were the ones that bit the most (41.05%) (1626/3961) (Figure 1), with a statistically significant difference between the different dog sizes (Chi-Square = 118.28; *p* = 0.001).

In terms of breed, mixed-breed dogs led the list with 55.99% (2298/4104). Followed by the German Shepherd (8.50%) (349/4104) and by the Poodle (7.29%) (299/4104) (Table 7). Furthermore, a significant difference between the different classification types of breeds of biting dogs (Potentially Dangerous Dogs (P.D.D), Non-Potentially Dangerous Dogs (N.P.D.D), Others and Mixed-breed) (Chi-Square = 3328; *p* = 0.001).

#### 3.2.3. Place of Residence of the Biting Animal

With regard to the place where the animal resided, two relevant variables associated with this point were considered. The first was the situation of ownership of the biting dog and the second if the animal lived with the victim.

Of the 6533 canine bite records, 100% (6533/6533) had information about whether the biting animal lived in the victim’s home. Of these, 50.07% (3271/6533) of the dogs did not live with the victim and a lower percentage (49.93%) (3262/6533) did live in the victim’s home. There was no significant difference between both records (Chi-Square = 0.012; *p* = 0.911) (Table 8).

There was an association between the variable “the biting animal lives in the victim’s house” and “the victim is the owner of the biting animal” (Chi-Square = 1821.53; *p* = 0.001). Victims who did not own the animal (the owner being the legal owner of the animal) and did not live with it, were more likely to be bitten than the rest of the people in this study (Table 9).

#### 3.2.4. Relationship of the Biting Animal and the Victim

Regarding the relationship between the biting animal and the victim, the highest percentage of the animals involved in this type of incident did not belong to the victims (74.51%) (4868/6533), only 25.49% (1665/6533) belonged to the person bitten (Chi-Square = 1570.37; *p* = 0.001).

### 3.3. Context of the Attack

#### 3.3.1. Location Where the Incident Occurred

A statistically significant difference (Chi-Square= 2157.36; *p* = 0.001) was found between all the categories of this variable. The highest number of attacks was registered within the compound or home (57.50%) (3755/6531) followed by public spaces (31.91%) (2084/6531) and finally others (10.60%) (692/6531) (Table 10).

The highest percentage of dog bite victims were attacked inside the home. In these cases, most of the biting dogs lived with the victims, but were not owned by them (22.53%) (1472/6533). On the other hand, the highest percentage of the people who were bitten on public roads did not own the dog and did not live with the biting animal (27.26%) (1781/6533) (Table 11).

When performing a chi-square test, an association was found between the place where the bite occurred and living with the biting dog (Chi-Square = 2317.30; *p* = 0.001). In particular, people who lived with the attacking dog presented a higher percentage of attacks within the home (42.77%) (2798/6533). In the same way, people who did not live with the biting dog were mostly bitten on the road or public space (27.46%) (1794/6533).

Finally, when classifying the age groups of victims according to the location of the incident, groups 4 and 5 were associated with a greater number of bites on the street or public space, whereas age groups 1, 2 and 7 presented a greater association with bites within a compound or a home (Chi-Square = 49.89; *p* = 0.001) (Table 12).

#### 3.3.2. Seasonality of the Attack

**Figure 2:**
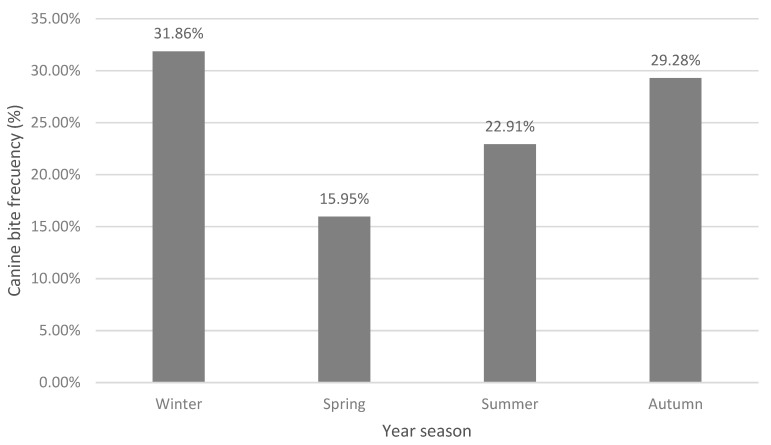
Distribution of bites according to the season of the year in which the incident occurred. When crossing the variables season in which the incident occurred, the place where it occurred and if the victim was the owner of the biting dog, it was observed that the highest number of incidents occurred in winter, within the home, and towards a person who did not own the aggressor animal (14.48%) (946/6533). Likewise, an association between location and season of the year was found (Chi-Square = 971.77; *p* = 0.001) (Table 13).

The results associated with the distribution of the attacks according to the season of the year in which the incident occurred showed a significant difference between the different periods of the year (Chi-Square = 394.45; *p* = 0.001), with the highest number of episodes being registered in winter (31.85%) (2081/6533), followed by autumn (28.28%) (1912/6533), summer (22.91%) (1496/6533) and finally spring (15.95%) (1044/6531) (Figure 2).

### 3.4. Characteristics of the Injury

#### Type of Bite

Most of the canine bites recorded in the present study were single bites (89.63%) (5598/6246), differing significantly (Chi-Square = 3922.91; *p* = 0.001) from the multiple ones (10.37%) (648/6246) (Table 14).

## 4. Discussion

In this study, we analyzed 17,299 incidents of bites registered in the “Bite Animal Registration System” (BARS) of the Chilean Ministry of Health. These data were recorded during the period 17 September 2017 and 17 September 2018. Although there are previous studies on this problem in the country [23,32], this is the first study with national coverage.

This research focused specifically on dog bites as these are the ones that occur most frequently, as indicated by previous studies in Chile [23,34] and in other countries such as Iran [35,36], Korea [37] and Bosnia and Herzegovina [38].

### 4.1. Data Collection Systems

This study analyzed 17,299 incidents of bites registered in the “System for the Registration of Chewing Animals” of the Ministry of Health of Chile, which corresponded to 100% (17,299/17,299) of the patients treated for this cause in the main public health services of primary and emergency care in the country, for one year.

Of these 17,299 records, only 41.74% (7220/17,299) had information on the biting animal, which made it possible to differentiate the dogs participating in these incidents for further analysis. This percentage was lower than that reported in previous studies carried out in Chile (96.30%) (79.5%) [23,34] and in Iran (99.98%) [35]. In contrast, the numbers were higher than those reported in a study in India, where only 8.18% of the records had this information [39]. Although the results of the present investigation were not as low as those mentioned in the study in India, they were well below those obtained in previous investigations carried out in Chile. This may be influenced by different factors associated with the use of the data collection digital system, for example, factors related to the person (age of the person in charge of recording the data, physical and cognitive condition and attitude towards technology), factors related to the task (how familiar is the user with the task or how complex is the technology to be used) and technological factors (the quality of internet connection, ease of accessing the information to be used, among others) [40]. It is important to consider these factors as, since 2017, the Chilean Ministry of Health has used a digital method to collect information of this nature, which differs from the manual method applied in the previously mentioned Chilean investigations. Thus, when considering them to explain why the low percentage of completion of information on the type of biting animal, they were discarded, as most of the variables considered in this article pertaining to general bites (61.53%) had a completion percentage greater than 94%. Therefore, the factor that must be influencing this poor recording of this particular variable must be based on some specific point in the way in which the information on this variable is being collected. Another factor that could influence the lack of information about the species of the biting animal could be the complexity of the existing scenarios in the emergency services, such as, noisy, hectic and crowded work environments, distractions of the relatives of those treated, insufficient space to carry out registration procedures, among others [41,42]. However, these factors should also not be fully responsible for the lack of information on the type of biting animal, as this should affect the completeness of all variables more equitably than just one. In addition, this factor was also ruled out, as if it existed it would have affected the studies carried out manually as well as our study that used the digital system. Finally, we consider that the safest factor could be the modification made in the design of the questions used to collect information on the BARS digital platform, which is different from that used in previous studies carried out on the same subject with sources from the Ministry of Health. This could justify the differences in the percentages of information gathering between the studies previously carried out in Chile and the one developed here.

Despite the limitations related to digital registration systems, for example, in the case of the question related to the type of biting animal, asking the same question twice and on one of those occasions leaving only the possibility of marking in the same box dog or cat, not being able to differentiate whether it is one or the other. In the other question related to the same variable, canines can be differentiated from cats, but many people who have already filled in the first box do not complete the latter. Therefore, valuable information for this differentiation is lost. However, it should be noted that digitization has been of great contribution to achieving the goals and action plans projected in the last decade by the Ministry of Health of Chile, which is currently focused on improving the quality of care for Chileans. This seeks to favour the characterization of the population and the phenomena that affect it from a public health point of view, as well as to improve the quality of information at the central level through the Modernization of Digital Information System of the Health Authority [43].

### 4.2. Region Where the Attack Occurred

Chile is administratively divided into 16 regions. Chile is administratively divided into 16 regions. However, at the time of the study, it was only divided into 15 regions, with which we are working in this research. When analysing the incidents by region, it was observed that the largest number of records of canine bites were concentrated in the Metropolitan region with 29.22% (1909/6533), and in the Valparaíso Region with 19.25% (1911/6533). Previous studies reported a similar situation, although it was the Valparaíso region that obtained the highest percentage of records, followed by the Metropolitan region [23]. In the present study, the sum of these two regions accounts for more than half of the bite incidents registered at the national level. This finding may be related to common characteristics of both regions, as they are the two most populated regions of the country and have a high percentage of urban population (Valparaíso: 91.01% and Metropolitan Region: 96.30%). In addition, the capital of Chile, Santiago, is located in the Metropolitan Region; while Valparaíso, the main port of the country, is located in the homonymous Region [33]. Finally, both regions have a high density of dogs on the streets. All these factors can directly influence the high numbers of animals that interact with people in confined spaces [22].

### 4.3. Background of the Biting Animal

#### 4.3.1. Type of Biting Animal

Of the total number of animal bite records with information (7220/17,299), 90.48% (6533/7220) were caused by dogs; followed by cats with 9.52% (687/7220). These numbers coincide with a previous Chilean study [23] in which the species responsible for the highest number of bites were dogs with 91.6% and then cats with 5.6% (281/5003). The same was reported by Villagra [34], who investigated this problem in Los Andes, Chile, also identifying the dog as the main cause of bites. Other studies in Chile report something similar [44]. The same situation is observed in other countries, for example, in Iran [36,45,46] and Korea [37].

These results are because dogs are the most common companion animals in Chile and in other countries such as the United States, where 38.2% of households have a dog as a pet [47]. In Chile, some publications indicate that 52% of households have a dog as a pet and 36% have a cat as a pet [48]. A high number of these animals are kept under poor supervision conditions, circulating freely on roads and public spaces, and it is reported that up to 67% of all owned dogs are temporarily abandoned [48].

#### 4.3.2. Size of the Biting Animal

In this study, it was observed that most of the recorded incidents involved medium-sized dogs. This could be because only those incidents of bites that required attention in emergency centres were analyzed, probably ruling out incidents caused by small animals, as due to their size and attack power, they produce less damage than larger animals.

The results of this study agree with those recorded by Buso [49], where the highest number of dogs involved in bite incidents in Brazil (Sao Paulo) were of medium size (46%). These results can be explained by different factors, including the severity of the injuries inflicted by the biting dog, as the larger the animal, the more likely it is to produce injuries that lead to medical consultation.

#### 4.3.3. Breed of the Biting Dog

The highest percentage of incidents were caused by mixed-breed dogs (55.99%) (2298/4104), the rest of the incidents were distributed in more than 73 breeds, among which the German Shepherd stands out (8.50%) (349/4104). These results agree with previous research on canine demography that shows that mixed-breed dogs are the most common in Chile and that they cause the majority of bites [34,50]. A study carried out in the city of Puerto Aysén, Chile, also found that bites were caused mainly by mixed-breed dogs (73.9%) (176/238), followed by the German Shepherd (7.1%) (17/238) [50]. Internationally, similar findings have been reported [34,51,52].

The frequency of participation of German Shepherd dogs in bites should be interpreted with caution, as they are one of the most frequent breeds in various countries, with similar findings reported in the United Kingdom [51] and the United States [52,53,54]. In Chile, the 12-year-old Kennel Club records show that it is the breed with the highest number of registered specimens, reaching 10.25% of the total [54]. On the other hand, the identification of the breed is carried out by the affected person, and the participation of the breed may be overestimated because it is the most common or due to lack of knowledge, including mixed-breed dogs or those of breeds with similar characteristics [55,56]. It is known that the precision of breed identification based on physical characteristics can lead to errors [57].

It is worth noting that, with regard to the classification of dogs by breed hazard classification, of the 73 participating breeds, only six breeds belonged to the P.D.D group and of these, the breed with the highest participation was the Pit Bull breed with only 2.02% (83/4104). These values are well below those recorded by the three first places of biting breeds (Mixed breed, German Shepherd and Poodle) who concentrate 71.78% (2946/4104). The two main breeds that participated in these episodes coincided with those obtained by [58] in Spain, where mixed breed and German Shepherds were the most frequent biters. It is also important to note that when analysing the ten breeds with the highest frequency of bites, this research as well as that conducted by Rosado [58], only includes a single breed considered P.D.D and in both articles with very low participation (2.02%) in this research and 2.1% in Rosado [58]. This low participation of P.D.D breeds coincided with that recorded by Oxley [52] who found that within the five main breeds involved in dog attacks in the UK, none belonged to the P.D.D group. These results may suggest that the P.D.D breeds should not be the main focus of prevention programs, as they would not be the ones that bite the most. In the case of Chile, the high participation of mixed-breed would complicate the enforcement of regulations focused on certain breeds in particular.

#### 4.3.4. Ownership Situation (the Biting Animal Has a Residence)

The largest number of biting dogs had a known residence. These results coincide with those obtained by [59] in Iran where most of the dogs that bit had an owner (92%). Likewise, they agree with a study carried out in the Metropolitan Region, where it was shown that 80.5% of the attacks were produced by animals with residence, of which 37.25% had total confinement by their owners and 43.25%, despite having owners, roamed freely on the streets [23]. These results highlight the precarious conditions for keeping animals in Chile, as evidenced by Ibarra [60], who recorded the high number of dogs that roam the street without restriction, which were divided into strays (with owner) (52.4%), homeless (no owner) (21.9%) or community-owned (no specific owner) (8.9%). It is therefore essential to consider the importance of dog population management, as it has a role to play in improving the health and welfare of free-roaming animals, controlling rabies, minimizing the problems associated with the free movement of such dogs [61,62] and reducing dog bites associated with uncontrolled street dog traffic [62]. Therefore, to successfully manage both dog bites and the rabies virus, an effective and cost-effective dog population control program, adapted to the reality of the intervention site, should be considered [61,62,63]. This can help minimize the risk of people being bitten by reducing uncontrolled interactions in public spaces and help stabilize dog populations by facilitating rabies vaccination coverage [61]. It is important to note that in this country, specifically in the Coquimbo region, vaccination levels for Distemper have been recorded at 29% and 30% against canine Parvovirus [22]. In another study conducted in the commune of Santiago, 78.6% of the dog population included in this research was recorded as having been vaccinated for rabies virus [21].

On the other hand, bite incidents can generate negative impacts on the relationship between people and animals, such as mistreatment, abandonment, even the elimination of such animals from the community without considerations of animal welfare [60], transgressing the concepts of One Health and One Welfare, which are essential for healthy and safe coexistence between dogs and humans. It is important to consider that the animals that are attacking in addition to directly biting people, can also direct their attack on other animals, damaging the environment and creating significant problems both in the community that feels threatened, as well as the owners of the animals affected (production animals) and wildlife that is attacked in the environment. Likewise, physical injuries and disease transmission occur between dogs and humans; and dogs and other animals, affecting their physical well-being. Furthermore, the very animals that bite are often abused and abandoned, damaging their own welfare [63]. All this problem is closely related to deficiencies in the responsible ownership of these dogs, a problem widely considered in this article. This will be reflected in well-being problems both in the environment, in people and animals, essential pillars in the concept of “One Welfare” [64]. Likewise, these episodes affect the health of both people and affected animals, facilitating the transmission of zoonoses, causing infections, physical and psychological injuries, fundamental factors in the concept of “One Health”.

#### 4.3.5. The Animal Lives with the Victim

Regarding whether or not the animal lived with the victim, in the present study no significant differences were found in this variable. However, victims who did not own the animal and did not live with it were more likely to be bitten than the rest of the people in this study (*p* = 0.001).

#### 4.3.6. The Dog Belonged to the Victim

The highest percentage of dogs involved in biting incidents did not belong to the victims (74.51%) (4868/6533), presenting a statistically significant difference with those that did. These results coincide with a study carried out in the United States [65] where the highest number of attacks were caused by unfamiliar dogs (41.1%), but they differ from those obtained by [60] who found that in Iran the highest number of biting animals belonged to the victim, followed by neighbourhood dogs. In the same way, in Chile, it was evidenced that the highest number of bites were made by animals owned by the victim (33.7%) [34].

Considering that in the present study most of the biting dogs did not belong to the victims but lived with them and the attack occurred inside the homes, it is most likely that the dogs belonged to people who lived with the victims, either from the same family, some other member of the family nucleus or people who shared the same living space. This result agrees with that obtained by Caffrey [66], where most of the severe incidents occurred within the dog’s home. Likewise, according to another study carried out in Iran, the highest percentage of bites occurred inside the house (58.5%); however, the majority of these attacks were directed at the owners of the dogs (26.6%) [67], unlike in the present investigation where the main victims were not owners of the biting dog. These results coincide with those obtained in Florida and the United States [65], where most of the attacks occurred in the home of the owner of the aggressor dog (53.4%) but the animal was unknown to the victim (41.4%), unlike the people bitten in this study. Two factors may have influenced the records of this research, the first is the irresponsibility of the dog owners, who often do not measure the injuries that their animals can produce at the time of an attack, nor do they know the main risk contexts. This leads to trust in how the animal interacts with the environment and is therefore often left unsupervised. This behaviour can increase when adults who live with the animal transfer responsibility to the person who is directly interacting with it. The second factor is that people who live with the animal build bonds that often make them omit necessary security measures with the animal, or do not know how to read the animal’s signals, which predisposes them to different types of attacks. It is important to understand that this scenario does not prevent some incidents from also occurring on the streets, as a result of irresponsible animal husbandry and the high number of animals that roam the streets of this country.

### 4.4. Victim’s Background

#### 4.4.1. Victim’s Gender

In relation to the victim’s gender, men were bitten more than women, which is consistent with previous studies in other countries, such as the United States [68,69], Korea [37], Bosnia and Herzegovina [38], Iran [69] and Ireland [70]; as well as with previous Chilean publications [23,34,44,71]. These results can be explained because men can have a more invasive type of approach with the animal, they can also be less cautious or have less perception of an eventual threat.

In the case of minors, the higher incidence of biting by male individuals compared to female individuals has been explained by a higher level of impulsiveness [72] and a greater interest in experiencing new sensations [73]. It was also observed that boys with greater curiosity and activity were at greater risk of being bitten [74]. This could be extrapolated to differences in the way adult males approach dogs compared to females, with the former approaching dogs in a more challenging and invasive manner, which may result in a potential bite incident.

#### 4.4.2. Victim’s Age

The age group with the highest number of bites was 40–64 years. This can be difficult to compare with other studies, as age stratification varies by author. The results of this study are close to those obtained in Bosnia and Herzegovina, where it was reported that the most bitten people were adults between 50–64 years and 25–49 years of age (n = 425, 24.7% and n = 390, 22.7%, respectively) [38]. In Chile, similar results have been reported, such as those found in the city of Los Andes, where the most bitten were people between 18 and 59 years of age [44].

A previous study reported that the highest number of bites occurred in children aged 5–9 years. This makes sense, as children of that age can have more invasive interactions by failing to understand the body language of dogs, which is essential to avoid biting incidents. Likewise, children in this age group are noisy and erratic in their movements, which can be intimidating for the dog with whom they interact [23].

A higher frequency of bites in adults than in children may be the result of public awareness, achieved thanks to the efforts of scientific societies and specialists in public health and ethology, regarding the importance of supervising the interaction between dogs and children [75,76]. An attempt has been made to strengthen the importance of reading the animal’s body language and respecting the message sent, especially by children. However, it is interesting that the number of bites does not seem to have decreased.

Although the importance of preventing this type of incident in children has become widespread in Chile and small sections have been developed in the country’s responsible possession material aimed at sending information to prevent bites in children [77] there is still much to be done. However, adults may have focused their attention on preventing these types of attacks on children and have neglected their own safety.

Finally, it may be interesting to consider the ageing of the Chilean population as an explanation for these results [20], which may be influencing the increased participation of adults in this type of incidents.

### 4.5. Context of the Attack

#### 4.5.1. Location Where the Event Occurred

Regarding the location where the incident occurred, a statistically significant difference (*p* = 0.001) was found between the areas classified in this variable. The highest number of attacks was registered within the compound or home (57.50%) (3755/6531). This coincides with multiple studies from developed countries, where there are important socio-cultural and ownership differences with Chile as in the latter there are behaviours that go against responsible ownership, such as having a high number of free-roaming dogs on the streets, unlike developed countries that do not present scenarios of this type. This is due, among other things, to the high percentages of dogs with owners who are allowed to roam freely on the streets (27% in cities and 67% in rural areas) [22]. An example of a developed country that produced similar results is a study of children in China where most attacks occurred within the home of the animal that bit it [74]. The same was evidenced in another study conducted in Philadelphia, where 52% of the incidents occurred indoors [78]. All these antecedents agree with what was stated by Caffrey [66], who proposed that the probability of a bite incident occurring at home was 8.17 times higher than in public places in Calgary, Canada. In this same study, the predicted probability of a high-severity incident was higher at home than in a park (with the dog off the leash), on the owner’s property, or in a public space. Additionally, the predicted probability of high-severity incidents occurring on the owner’s property or in a park was statistically higher than that of public spaces.

In contrast, in less developed countries, such as India, bite incidents occur mainly on the street, by stray dogs, while people walk without prior provocation of the animal [79]. The high number of dogs roaming the streets without supervision, which predisposes to biting incidents, is also seen in Chile, where high percentages of stray dogs (with an owner) (52.4%), homeless (without an owner) (21.9%) or community (without specific owner) (8.9%) have been registered [60]. This could support the results of previous studies in Chile [21,80,81,82] where the highest number of canine bites occurred in the street (62.1%, 64%, 73.3% and 69.2% in the different studies).

Despite the scenario described above, the present investigation found opposite results in relation to the location of the attack. This may be the result of certain motivations and pet ownership habits that the Chilean population presents today, which have been modified over the years, and may have influenced the change in the results associated with canine bites. An example of this change is the reason for adopting a dog, in a study carried out 17 years ago [24] in the Metropolitan Region of Chile, most of the adopted dogs were incorporated into homes for a protective motivation (41.1%). However, more recent studies have reported a greater motivation for affective or companion adoption, as described by Morales [24], who recorded the effective reason as the main cause of adoption (86.9%). It is important to mention that these comparisons have been made on articles from the same region, as depending on the location evaluated, the results may vary due to other factors. In addition, the Metropolitan region has the highest number of bite incidents, a problem that we will try to explain. The relationship with a companion pet could influence the habits of interaction with animals, which could facilitate the occurrence of bites, all associated with close contact. In a 2019 Chilean study, 90% of the pet owners interviewed indicated that their companion animals lived totally or partially inside the house. In this work, 56% of the interviewees said that their dogs lived only indoors and 34% inside and outside the house. In addition, they mentioned that the dogs slept inside the owner’s bed (34%) [83]. All these interaction patterns of high proximity inside the home may be a sign of other patterns that could be influencing the high number of attacks that occurred in this location. These changes may be related to changes in both economic and human development indicators in recent decades. For example, the Human Development Index (HDI-UNDP), the year 2003 = 0.774, high level [84] and for the year 2019 was 0.847, very high level [19]. This brings it closer to the characteristics of developed countries, but still in transition. This will be seen in changes in animal interaction habits gradually. All this may also affect the dynamics of dog bite incidents in Chile.

Another factor that can influence these results is the high number of animals that live in confined spaces, sharing with their family nucleus more closely and thus predisposing people around them to suffer bites. These characteristics of coexistence between dogs and humans favour the potential interaction between these individuals in contexts that can trigger different types of aggression, such as aggression due to pain, aggression due to fear, and redirected aggression, among others [85,86]. An example of this is the study carried out by Morales [21] in the commune of Santiago where most of the dogs live in apartments without a patio (48.3%). This is interesting, as previous studies [48,87] have considered that urban environments, where homes are smaller, with more limited access to green areas and longer periods of indoor permanence and isolation, can influence the increase in dog bites, compared to rural homes where the situation is different.

On the other hand, the average number of people per household in Chile was 3.1 in 2017 [88]. This value, which is higher in households with high or critical overcrowding indexes where more than five people live, corresponds to 4.7% of Chilean households, both in urban and rural areas [88], being the Metropolitan Region that with the second-highest percentage (8.7%). Likewise, in Chile, “*allegamiento*” is a “*strategy used by households and family nuclei to solve homelessness, sharing a home with another household or nucleus*” [88]. This type of housing is concentrated in the poorest quintiles of the population [88], the socioeconomic stratum that has the highest percentage of pet ownership (69%) [48]. All the conditions previously described expose a reality that is important to consider to explain the high number of reports of canine bites within homes, specifically to people who live with the animal but do not own it, as although the owner of a dog may have the education and disposition to interact safely with his companion animal, the rest of the people who live with it do not necessarily have the same characteristics. This scenario can be complicated by the reduced spaces and by living with a high number of people.

To date, most Chilean studies have reported that the places where the bite incident occurred the most were similar to those reported in developing or underdeveloped countries, especially due to the large number of dogs that roam freely in the streets. An example of this is a study conducted in the Metropolitan region, where it was found that the highest number of bites occurred on the street (77.2%) and generally by dogs with owners but that roamed the streets. Fourteen years after presenting these results, this study shows that the places where the bite episodes occur with greater frequency have changed. This may be influenced by changes in animal ownership realities that have occurred in Chile, which do not necessarily differ in the levels of responsibility towards companion animals but may indicate a transition to certain behaviours in relation to human-dog interaction, similar to developed countries. It is interesting to understand that Chile’s level of development is evolving, as in recent decades there has been growth towards higher levels of development, which can be seen, for example, in the Human Development Index (HDI-UNDP), which for the year 2003 was 0.774, high level, rank no. 45; and for 2019 it was 0.847, very high level, rank no. 42. The same is suggested by the OECD indicator of scientific performance (PISA), which obtained an average score of 448 in 2006, and 444 in 2018; and the World Bank indices (GDP year 2003: US$ per capita: 4772,563, GINI Inequality Index 2003: 51.5; GDP 2018 US$ per capita: 15,923.3, GINI Inequality Index 2017: 44.4). It is important to mention that despite having these good development indices, the variables used to calculate these indicators are not always directly related to pet ownership habits and it should also be taken into account that they do not fully reflect the reality of the country, as for example GDP per capita is altered by the levels of inequality in Chile, which would directly influence how people interact with animals (due to levels of overcrowding, hidden poverty and others). Therefore, the results that should be considered to determine the context of the bite incidents should be evaluated with variables raised specifically for this topic and use the country’s general development indicators only as a compliment.

#### 4.5.2. Seasonality

The highest number of canine attacks was registered in winter and autumn, which differs from most of the previous studies in the country, where a higher frequency of incidents was reported in summer [34,44,71]. It also differs from international publications, where a higher concentration of bites is reported in spring [38] or summer [52]. This could be because nowadays people in Chile are closer to their dogs than a few decades ago; before the dog spent most of the time outside the house, while now it spends more time inside it and is treated as one of the family. This habit can increase in colder seasons, and it can predispose people to more bite incidents.

#### 4.5.3. Type of Bite

In the present study, most of the canine bites were single (89.63%) (5598/6246), differing significantly (*p* = 0.001) from the multiple ones (10.37%) (648/6246). This is consistent with previous studies conducted in the same country [23,34]. The first of these studies included the regions of Arica, Coquimbo, Metropolitan, Valparaíso, Bío Bío and Aysén, finding that 86.58% of the bites were single. Likewise, a study in the United Kingdom reported that 86% of bites were single [52].

## 5. Conclusions

In total, 17,299 bite incidents were recorded in the period under review (September 2017 to September 2018).

The highest number of bites was recorded in the most densely populated regions of the country, the Metropolitan Region and the Valparaiso Region. Most accidents were caused by mixed-breed and medium-sized dogs. Autumn and winter were the seasons with the highest percentage of reported incidents.

Two broad scenarios can be defined in terms of the location where the accident took place and the relationship between the victim and the dog. Most biting episodes occurred inside the house and were directed to family-members, whereas accidents in public places mainly affected non-family members.

To develop effective preventive strategies for dog bites, it is important to have a good understanding of the various demographic, situational and socio-cultural risk factors in these episodes. The results of our study could give insights on the different scenarios and risk factors that should be taken into account in the development of more efficient and cost-effective educational and regulatory interventions for the prevention of dog bites.

It is essential to incorporate the One Health approach in the prevention and control measures of this problem, focused mainly on the promotion of habits and customs to enhance the human-dog bond and the positive daily interactions between these animals and the people around them. It is also important to promote the welfare and health of dogs, people and the environment.

## 6. Limitations of the Study

This study used a secondary source of information, in which biting incidents are recorded to monitor rabies, so the analysis was adjusted to the pre-established collection format. On the other hand, not all the information fields were completed at the time of recording an incident, with a large number of cases missing relevant information, such as the registration of the biting animal. Furthermore, interesting data from an epidemiological point of view were not included, such as, for example, whether the incident occurred in an urban or rural area, which constitutes important information for the comprehensive understanding and confrontation of the problem [83].

On the other hand, taking into account the purpose of the database, there was no detailed information on the context of the attack, which would have been very useful to better understand the trigger agents, which could provide elements for the elaboration of prevention programs and public policy decision-making focused on controlling this important public health problem.

In addition, in the bite location item, when incorporating the alternative of another, valuable information is lost. Therefore, it should allow you to limit the answer to a more specific form of a question.

The database used does not contemplate bite incidents with a fatal outcome, because they escape the purpose of this registry. These types of incidents are registered by another government institution (Medical Legal Service).

Finally, a factor to improve in future research would be to have the total number of people treated in the corresponding emergency services, to make a comparative analysis between the total number of people treated in that place and the people treated specifically for canine bites.

## Figures and Tables

**Table 1:** Classification of the variables of interest for subsequent analysis.

Variable	Variable Description	Variable Classification
Victim’s Background		
Gender	Biological sex of the bitten person.	Man Woman
Age group	Age group of the victim, measured in years.	Group 1 (0–4 years) Group 2 (>4–9 years) Group 3 (>9–14 years) Group 4 (>14–25 years) Group 5 (>25–40 years) Group 6 (>40–64 years) Group 7 (>64 years)
**Information about the Biting Animal**		
Relationship of the victim to the biting animal	“Ownership” refers to the legal owner of the animal.	The dog belonged to the victim The dog did not belong to the victim Not reported
The biting animal lives in the victim’s home	“lives with the victim” refers to any dog that coexists with the victim in the same household, even if they go free roaming during the day.	Yes No
Species	Species of the animal involved in the bite incident	Canine Feline Other
Size	Subjective statement of the affected person in relation to the size or height of the biting animal	Small Medium Large
Breed	The breeds declared in the registers of biting animals were considered, discounting animals of mixed breeds, as it is a mixture of breeds. The classification of “Other” corresponds to the animals that the victims could not recognise as being of a certain breed.	Mixed-breed German Shepperd Poodle Fox Terrier Dachshund American Pit Bull Terrier Other Labrador Retriever Boxer Akita Cocker Spaniel Rottweiler Beagle Golden Retriever Yorkshire Terrier Chinese Shar-Pei Saint Bernard Bulldog Maltese Chow Chow Galgo Chihuahua Bull Terrier Pug Poodle Toy Belgian Shepperd English Shepperd French Bulldog Great Dane Fox Terrier Toy Shih Tzu Siberian Husky Collie Neapolitan Mastiff Samoyed Weimaraner Border Collie Argentine Dogo Miniature Schnauzer Basset Hound Pekingese Miniature Yorkshire Terrier West Highland White Terrier (West Highland) Bracco Italiano Dalmatian Australian Shepperd Pointer Bernese Mountain dog Cane Corso French Mastiff English Cocker Spaniel (Cocker spaniel) Galgo Italiano Labradoodle Pocket Beagle Puggle Affenpinscher Alaskan Malamute Black Russian Terrier Border Terrier Boston Terrier Bullmastiff Cardigan Welsh Corgi Coton de Tulear Doberman Pinscher (Doberman) Great Pyrenees Japanese Chin Lhasa Apso Mastiff Belgian Malinois Anatolian Shepperd Schipperke Standard Schnauzer Giant Schnauzer Australian Terrier
Classification of breeds according to “dangerousness”	The breeds included as Potentially Dangerous Dogs (P.D.D) have been considered, following those incorporated in the Law 21.020 of Responsible Tenure of Chile [29] and the Law 50/1999 of Potentially Dangerous Dogs of Spain [30]. The remaining breeds are Not Potentially Dangerous Dog (N.P.D.D), the variable “Others” the animals in which the breed was not identified and Mixed Breed, those that do not belong to pure breeds.	Potentially Dangerous Dogs (P.D.D) Non-Potentially Dangerous Dogs (N.P.D.D) Mixed-breed Other
Ownership status	Classification of the biting animal according to whether or not it had a known address (This does not mean that it has an owner)	Does not have a residence Has a residence
**Information about the Context of the Attack**		
Seasonality of the attack Factors that influence the location of the bite.	Time of year the bite incident occurs Site where the bite incident occurs	Spring Summer Autumn Winter Inside compound or home On public road or spaces Other
**Information on the Characteristics of the Injury**		
Type of bite.	Number of bites per victim	Single Multiple

**Table 2:** Distribution of total records according to the species of the biting animal.

Species of the Biting Animal	Frequency (n)	Frequency (%)
Canine	6533	37.77
Feline	687	3.97
No information	10,079	58.26
Total	17,299	100%

**Table 3:** Frequency of total canine bites by regions of Chile.

Region	Region Number	Bite Frequency (n)	Bite Frequency (%)	Total Human Population *	Total Dog Population **	Per Capita Incidence of Bites
Arica y Parinacota	15	97	1.48%	241,901	52,587	0.00040
Tarapacá	1	74	1.13%	354,94	77,161	0.00208
Antofagasta	2	278	4.26%	645,022	140,222	0.00043
Atacama	3	323	4.94%	307,835	66,921	0.00105
Coquimbo	4	168	2.57%	807,213	175,481	0.00021
Valparaíso	5	1911	29.25%	1,910,385	415,301	0.00100
Metropolitana de Santiago	13	1909	29.22%	7,702,891	1,674,542	0.00025
Libertador General Bernardo O’higgins	6	790	12.09%	966,486	210,106	0.00082
Maule	7	144	2.20%	1,105,731	240,376	0.00013
Biobío	8	122	1.87%	2,149,708	467,328	0.00006
La Araucanía	9	460	7.04%	1,001,420	217,7	0.00459
Los Ríos	14	68	1.04%	400,935	87,16	0.00017
Los Lagos	10	43	0.66%	877,348	190,728	0.00005
Aysén del general Carlos Ibáñez del campo	11	37	0.57%	106,023	23,048	0.00035
Magallanes y de la antártica chilena	12	109	1.67%	152,394	33,129	0.00072
Total		6533	100%	18,730,232	4,071,790	

* [32]. ** [33].

**Table 4:** Distribution of canine bites by the victim’s age and the victim’s gender.

Victim’s Gender	Frequency (n)	Frequency (%)
Female	3125	47.85% ^a^
Male	3406	52.15% ^b^
Total	6531	100.00%

a and b indicate significant differences (Chi-Square = 12.0902; *p* = 0.001).

**Table 5:** Distribution of dog bites by age group of the victim.

Age Group	Rate 10,000 (Inhabitants)	Female	Female	Male	Male	Chi-Square	*p*-Value **	Total (n)	Total (%)
Group 1	0.39	299	4.83% ^a^	432	6.97% ^b^			731	11.80%
(0–4 years)						241.984	0.001		
Group 2	0.42	318	5.13% ^a^	476	7.68% ^b^			794	12.81%
(>4–9 years)						314.408	0.001		
Group 3	0.29	213	3.44% ^a^	328	5.29% ^b^			541	8.73%
(>9–14 years)						244.455	0.001		
Group 4	0.48	432	6.97% ^a^	477	7.70% ^a^			909	14.67%
(>14–25 years)						222.772	0.0.136		
Group 5	0.52	460	7.42% ^a^	513	8.28% ^a^			973	15.70%
(>25–40 years)						288.695	0.089		
Group 6	0.82	836	13.49% ^a^	706	11.39% ^b^			1542	24.89%
(>40–64 years)						109.598	0. 001		
Group 7	0.38	412	6.65% ^a^	294	474% ^b^			706	11.39%
(≥ 65 years)						197.224	0.001		
Total	3.48	2,97	47.93%	3226	52.07%	105.771	0.001	6196	100.00%

a and b indicate significant differences (*p* = 0.001). ** Male and female comparison.

**Table 6:** Frequency of bites by Region according to the biting animal species.

Region	Canine (n)	Canine (%)	Feline (n)	Feline (%)	Total (n)	Total (%)
Tarapacá	74	1.02%	10	0.14%	84	1.16%
Antofagasta	278	3.85%	34	0.47%	312	4.32%
Atacama	323	4.47%	33	0.46%	356	4.93%
Coquimbo	168	2.33%	13	0.18%	181	2.51%
Valparaíso	1911	26.47%	200	2.77%	2111	29.24%
Libertador General Bernardo O’higgins	790	10.94%	44	0.61%	834	11.55%
Maule	144	1.99%	11	0.15%	155	2.15%
Biobío	122	1.69%	9	0.12%	131	1.81%
La Araucanía	460	6.37%	36	0.50%	496	6.87%
Los Ríos	68	0.94%	6	0.08%	74	1.02%
Los Lagos	43	0.60%	1	0.01%	44	0.61%
Aysén del general Carlos Ibáñez del campo	37	0.51%	3	0.04%	40	0.55%
Región Metropolitana	1909	26.44%	264	3.66%	2173	30.10%
Magallanes y de la antártica chilena	109	1.51%	6	0.08%	115	1.59%
Arica y Parinacota	97	1.34%	17	0.24%	114	1.58%
Total	6533	90.48%	687	9.52%	7220	100.00%

**Table 7:** Distribution of canine bites by breed.

Classification of Breeds According to “Dangerousness”	Breed	Frequency (n)	Frequency (%)
**Mixed Breed**	Mixed-breed	2298	55.99%
**Non-Potentially Dangerous** **Dogs (N.P.D.D)**	German Sheperd	349	8.50%
	Poodle	299	7.29%
	Fox Terrier	106	2.58%
	Dachshund	92	2.24%
	Labrador Retriever	70	1.71%
	Boxer	61	1.49%
	Cocker Spaniel	41	1.00%
	Beagle	38	0.93%
	Golden Retriever	38	0.93%
	Yorkshire Terrier	38	0.93%
	Chinese Shar-Pei	34	0.83%
	Saint Bernard	33	0.80%
	Bulldog	31	0.76%
	Maltese	28	0.68%
	Chow Chow	26	0.63%
	Galgo	22	0.54%
	Chihuahua	17	0.41%
	Bull Terrier	16	0.39%
	Pug	14	0.34%
	Poodle Toy	13	0.32%
	Belgian Sheperd	12	0.29%
	English Sheperd	12	0.29%
	French Bulldog	11	0.27%
	Great Dane	11	0.27%
	Fox Terrier Toy	10	0.24%
	Shih Tzu	8	0.19%
	Siberian Husky	8	0.19%
	Collie	7	0.17%
	Neapolitan Mastiff	7	0.17%
	Samoyed	7	0.17%
	Weimaraner	7	0.17%
	Border Collie	6	0.15%
	Miniature Schnauzer	6	0.15%
	Basset Hound	5	0.12%
	Pekingese	5	0.12%
	Miniature Yorkshire Terrier	5	0.12%
	West Highland White Terrier (West Highland)	4	0.10%
	Bracco Italiano	3	0.07%
	Dalmatian	3	0.07%
	Australian Sheperd	3	0.07%
	Pointer	3	0.07%
	Bernese Mountain dog	2	0.05%
	Cane Corso	2	0.05%
	French Mastiff	2	0.05%
	English Cocker Spaniel (Cocker spaniel)	2	0.05%
	Galgo Italiano	2	0.05%
	Labradoodle	2	0.05%
	Pocket Beagle	2	0.05%
	Puggle	2	0.05%
	Affenpinscher	1	0.02%
	Alaskan Malamute	1	0.02%
	Black Russian Terrier	1	0.02%
	Border Terrier	1	0.02%
	Boston Terrier	1	0.02%
	Cardigan Welsh Corgi	1	0.02%
	Coton de Tulear	1	0.02%
	Great Pyrenees	1	0.02%
	Japanese Chin	1	0.02%
	Lhasa Apso	1	0.02%
	Mastiff	1	0.02%
	Belgian Malinois	1	0.02%
	Anatolian Sheperd	1	0.02%
	Schipperke	1	0.02%
	Standard Schnauzer	1	0.02%
	Giant Schnauzer	1	0.02%
	Australian Terrier	1	0.02%
Potentially Dangerous Dogs (P.D.D)	American Pit Bull Terrier	83	2.02%
	Akita	58	1.41%
	Rottweiler	39	0.95%
	Argentine Dogo	6	0.15%
	Bullmastiff	1	0.02%
	Doberman Pinscher (Doberman)	1	0.02%
Other	Other	76	1.85%
	Total	4104	100%

**Table 8:** Distribution of bites according to whether the biting animal shared a home with the victim.

The Biting Animal Lived in the Victim’s Home	Frequency (n)	Frequency (%)
No	3271	50.07%
Yes	3262	49.93%
Total	6533	100.00%

**Table 9:** Distribution of canine bites according to whether the biting dog lives with the victim and whether the victim is the owner of the dog.

	The Victim Is Owner of the Biting Dog					
The Biting Dog Lives with the Victim	No	No	Yes	Yes	Total	Total
No	3189	48.81%	82	1.26%	3271	50.07%
Yes	1679	25.70%	1583	24.23%	3262	49.93%
Total	4868	74.51%	1665	25.49%	6533	100.00%

**Table 10:** Distribution of canine bites according to the location of the attack.

Location Where the Attack Occurred	Frequency (n)	Frequency (%)
Inside compound or home	3755	57.48%
On public roads or spaces	2084	31.90%
Other	694	10.60%
Total	6533	100.00%

**Table 11:** Distribution of canine bites according to the location of the attack, ownership of the biting dog, and whether the dog lives with the victim.

	Not the Owner			Owner			Not the Owner			Owner				
Location	Does Not Live at Home	Lives at Home	Total Not the Owner	Does Not Live at Home	Lives at Home	Total Owner	Does Not Live at Home	Lives at Home	Total Not the Owner	Does Not Live at Home	Lives at Home	Total Owner	Total (n)	Total (%)
Inside compound or home	935	**1472**	2407	26	1322	1348	14.31%	**22.53%**	36.84%	0.40%	20.24%	20.63%	3755	57.48%
On public road or spaces	**1781**	160	1941	13	130	143	**27.26%**	2.45%	29.71%	0.20%	1.99%	2.19%	2084	31.90%
Other	473	47	520	43	131	174	7.24%	0.72%	7.96%	0.66%	2.01%	2.66%	694	10.62%
General total	3189	1679	4868	82	1583	1665	48.81%	25.70%	74.51%	1.26%	24.23%	25.49%	6533	100.00%

**Table 12:** Frequency of canine bites, according to the location where the incident occurred.

Age Group	Inside Compound or Home	In the Street or Public Spaces	Other	Inside Compound or Home	In the Street or Public Spaces	Other	Total	Total
Group 1 (0–4 years)	482	179	70	7.78%	2.89%	1.13%	731	11.80%
Group 2 (>4–9 years)	477	235	82	7.70%	3.79%	1.32%	794	12.81%
Group 3 (>9–14 years)	287	180	74	4.63%	2.91%	1.19%	541	8.73%
Group 4 (>14–25 years)	510	305	94	8.23%	4.92%	1.52%	909	14.67%
Group 5 (>25–40 years)	521	364	88	8.41%	5.87%	1.42%	973	15.70%
Group 6 (>40–64 years)	867	504	171	13.99%	8.13%	2.76%	1542	24.89%
Group 7 (≥65 years)	421	211	74	6.79%	3.41%	1.19%	706	11.39%
Total	3.565	1.978	653	57.54%	31.92%	10.54%	6196	100.00%

**Table 13:** Distribution of canine bites by season/ location, according to whether the victim was the owner of the biting animal.

	The Victim Is the Owner of the Biting Animal					
Season/Location	No	Yes	No	Yes	Total (n)	Total (%)
**Winter**	**1656**	**425**	**25.35%**	**6.51%**	**2081**	**31.85%**
Inside compound or home	946	388	14.48%	5.94%	1334	20.42%
On public road or spaces	608	32	9.31%	0.49%	640	9.80%
Other	102	5	1.56%	0.08%	107	1.64%
**Autumn**	**1561**	**351**	**23.89%**	**5.37%**	**1912**	**29.27%**
Inside compound or home	774	321	11.85%	4.91%	1095	16.76%
On public road or spaces	695	27	10.64%	0.41%	722	11.05%
Other	92	3	1.41%	0.05%	95	1.45%
**Spring**	**655**	**389**	**10.03%**	**5.95%**	**1044**	**15.98%**
Inside compound or home	216	220	3.31%	3.37%	436	6.67%
On public road or spaces	207	18	3.17%	0.28%	225	3.44%
Other	231	150	3.55%	2.31%	381	5.86%
**Summer**	**996**	**500**	**15.25%**	**7.65%**	**1496**	**22.90%**
Inside compound or home	471	419	7.21%	6.41%	890	13.62%
On public road or spaces	431	66	6.60%	1.01%	497	7.61%
Other	94	15	1.44%	0.23%	109	1.67%
Total	4868	1664	74.51%	25.49%	6531	100.00%

**Table 14:** Distribution of canine bites according to the type of bite.

Type of Bite	Frequency (n)	Frequency (%)
Multiple	648	10.37%
Single	5598	89.63%
Total	6246	100.00%

## Data Availability

Not applicable.

## References

[1] Ponsich A., Goutard F., Sorn S., Tarantola A. (2016). A prospective study on the incidence of dog bites and management in a rural Cambodian, rabies-endemic setting. Acta Trop..

[2] Cornelissen J.M., Hopster H. (2010). Dog bites in The Netherlands: A study of victims, injuries, circumstances and aggressors to support evaluation of breed specific legislation. Vet. J..

[3] Lyu C., Jewell M.P., Piron J., Ehnert K., Beeler E., Swanson A., Smith L.V., Kuo T. (2016). Burden of bites by dogs and other animals in Los Angeles County, California, 2009–2011. Public Health Rep..

[4] Ishaya T.S., Ibironke O.C., Stella I.E., Olatunde A.B., Gyang M.D., Israel B.J., Saidu J.A., Gambo R.A., Peterside K.R., Christianah A. (2016). Dog Bites and Rabies: A Decade Perspective in Nigeria (2005–2014). World Vet. J..

[5] Damborg P., Broens E., Chomel B., Guenther S., Pasmans F., Wagenaar J., Weese J., Wieler L., Windahl U., Vanrompay D. (2016). Bacterial Zoonoses Transmitted by Household Pets: State-of-the-Art and Future Perspectives for Targeted Research and Policy Actions. J. Comp. Pathol..

[6] Audu S., Mshelbwala P., Jahun B., Bouaddi K., Weese J. (2019). Two fatal cases of rabies in humans who did not receive rabies postexposure prophylaxis in Nigeria. Clin. Case Rep..

[7] Dedy N.J., Coghill S., Chandrashekar N.K.S., Bindra R.R. (2016). Capnocytophaga canimorsus sepsis following a minor dog bite to the finger: Case report. J. Hand Surg..

[8] Talley P., Snippes-Vagnone P., Smith K. (2014). Invasive Pasteurella multocida Infections–Report of Five Cases at a Minnesota Hospital, 2014. Zoonoses Public HLTH.

[9] Morgan M., Palmer J. (2007). Dog bites. BMJ.

[10] O’Brien D.C., Andre T.B., Robinson A.D., Squires L.D., Tollefson T.T. (2015). Dog bites of the head and neck: An evaluation of a common pediatric trauma and associated treatment. Am. J. Otolaryngol..

[11] Favi M., Rodriguez L., Espinosa C., Yung V. (2008). Rabia en Chile: 1989–2005. Rev. Chil. Infectol..

[12] Alegria-Moran R., Miranda D., Barnard M., Parra A., Lapierre L. (2017). Characterization of the epidemiology of bat-borne rabies in Chile between 2003 and 2013. Prev. Vet. Med..

[13] Instituto de Salud Pública Sección Rabia. http://www.ispch.cl/seccion-rabia.

[14] Ovalle R., Junod T. (2014). Análisis retrospectivo de la situación de vacunación antirrábica canina en Chile entre los años 2002 y 2012. Revista Chilena de Salud Pública.

[15] Ministerio de Salud Reglamento de Prevención y Control de la Rabia en el Hombre y en los Animales. https://www.bcn.cl/leychile/navegar?idNorma=1058839.

[16] De la Puente-León M., Levy M.Z., Toledo A., Recuenco S., Shinnick J.E., Castillo-Neyra R. (2020). Spatial inequality hides the burden of dog bites and the risk of dog-mediated human rabies. medRxiv.

[17] Georges K., Adesiyun A. (2008). An investigation into the prevalence of dog bites to primary school children in Trinidad. BMC Public Health.

[18] (2020). Organización para la Cooperación y el Desarrollo Económico (OECD). https://data.oecd.org/chile.htm.

[19] Conceição P. (2019). Human Development Report 2019. Beyond Income, Beyond Averages, Beyond Today: Inequalities in Human Development in the 21st Century.

[20] American Veterinary Medical Association Pet Ownership & Demographic. https://www.avma.org/resources-tools/reports-statistics/us-pet-ownership-statistics.

[21] Morales Fortuzzi R.I. (2017). Demografía de la población de perros (Canis familiaris), de las viviendas de la comuna de Santiago de Chile. http://repositorio.uchile.cl/handle/2250/151138.

[22] Acosta-Jamett G., Cleaveland S., Cunningham A.A., Bronsvoort B.D. (2010). Demography of domestic dogs in rural and urban areas of the Coquimbo region of Chile and implications for disease transmission. Prev. Vet. Med..

[23] Barrios C.L., Vidal M., Parra A., Valladares C., González C., Pavletic C. (2018). Epidemiological characterization of bites: A retrospective study of dog bites to humans in Chile during 2009. J. Vet. Behav..

[24] Ibarra L., Morales M.A., Cáceres L. (2003). Mordeduras a personas por ataque de perros en la ciudad de Santiago, Chile. Avances en Ciencias Veterinarias.

[25] Kitala P., McDermott J., Kyule M., Gathuma J., Perry B., Wandeler A. (2001). Dog ecology and demography information to support the planning of rabies control in Machakos District, Kenya. Acta Trop..

[26] Ngugi J.N., Maza A.K., Omolo O.J., Obonyo M. (2018). Epidemiology and surveillance of human animal-bite injuries and rabies post-exposure prophylaxis, in selected counties in Kenya, 2011–2016. BMC Public Health.

[27] Rothe K., Tsokos M., Handrick W. (2015). Animal and human bite wounds. Dtsch Ärztebl Int..

[28] Ministerio de Salud de Chile (2017). Sistema de Registro de Animales Mordedores.

[29] Ministerio del Interior y Seguridad Pública de Chile (2017). Reglamento que establece la forma y condiciones en que se aplicarán las normas sobre tenencia responsable de mascotas y animales de compañía y determina las normas que permitirán calificar a ciertos especímenes caninos como potencialmente peligrosos. Diario Oficial de La República de Chile.

[30] Gobierno de España Real Decreto 287/2002. https://www.boe.es/buscar/doc.php?id=BOE-A-2002-6016.

[31] Ministerio Secretaría General de la Presidencia de Chile Ley Sobre Protección de la Vida Privada. Ley 19628. https://www.bcn.cl/leychile/navegar?idNorma=141599.

[32] Instituto Nacional de Estadísticas Estimaciones y Proyecciones de la Población de Chile 1992–2050. http://www.censo2017.cl/descargas/proyecciones/metodologia-estimaciones-y-proyecciones-de-poblacion-chile-1992-2050.pdf.

[33] Morales M.A., Varas C., Ibarra L. (2009). Caracterización demográfica de la población de perros de Viña del Mar, Chile. Arch. Med. Vet..

[34] Villagra V., Cáceres D., Alvarado S., Salinas E., Caldera M.L., Lucero E., Vivani P., Torres M. (2017). Caracterización epidemiológica de mordeduras en personas, según registro de atención de urgencia: Provincia de Los Andes, Chile. Rev. Chil. Infectol..

[35] Havasian M.R., Rooghani A.L., YasemiI M.R., Rointan R., Hosseini R.A., Panahi J.A. (2015). Epidemiology of animal bites in region of Ilam, Iran. Organization.

[36] Dehghani R., Sharif A., Madani M., Kashani H.H., Sharif M.R. (2016). Factors influencing animal bites in Iran: A descriptive study. Osong Public Health Res. Perspect..

[37] Lee K.J., You Y., Kim Y.W., Lee D.C., Koh S.H., Kim J.S., Roh S.Y., Hong M.K. (2019). Domestic dog and cat bites: Epidemiology and analysis of 823 cases over the last 5 Years. J. Wound Manag. Res..

[38] Uzunović S., Skomorac M., Bašić F., Mijač-Musić I. (2019). Epidemiological features of human cases after bites/scratches from rabies-suspected animals in Zenica-Doboj Canton, Bosnia and Herzegovina. J. Prev. Med. Public Health.

[39] Venkatesan M., Dongre A.R., Kalaiselvan G. (2014). An epidemiological study of animal bites and envenomings in a rural district of Tamilnadu, India. J. Health Allied Sci..

[40] Benavides C.O. (2017). Perspectivas y retos en factores humanos e ingeniería de sistemas en ambientes médicos complejos. Acta Colombiana de Cuidado Intensivo.

[41] DeLucia P.R., Ott T.E., Palmieri P.A. (2009). Performance in nursing. Rev. Hum. Factors Ergon..

[42] Gurses A.P., Carayon P. (2007). Performance obstacles of intensive care nurses. Nurs. Res..

[43] Ministerio de Salud de Chile. http://www.salud-e.cl/prensa/la-digitalizacion-de-la-informacion-para-el-fortalecimiento-de-la-autoridad-sanitaria/.

[44] Salas Ramírez R., Villagra Castillo V., Torres Hidalgo M. (2019). Caracterización clínico-epidemiológica de mordeduras en personas mayores en la provincia de los Andes, región de Valparaíso, Chile. ARS Med..

[45] Babazadeh T., Nikbakhat H.A., Daemi A., Yegane-Kasgari M., Ghaffari-Fam S., Banaye-Jeddi M. (2016). Epidemiology of acute animal bite and the direct cost of rabies vaccination. J. Acute Dis..

[46] Esmaeilzadeh F., Rajabi A., Vahedi S., Shamsadiny M., Ghojogh M.G., Hatam N. (2017). Epidemiology of animal bites and factors associated with delays in initiating post-exposure prophylaxis for rabies prevention among animal bite cases: A population-based study. J. Prev. Med. Public Health.

[47] Burns K. Pet Ownership Stable, Veterinary Care Variable. https://www.avma.org/javma-news/2019-01-15/pet-ownership-stable-veterinary-care-variable.

[48] Adimark. Microestudio gkf: Los chilenos y sus mascotas. https:/cdn2.hubspot.net/hubfs/2405078/cms-pdfs/fileadmin/user_upload/country_one_pager/cl/gfk_los_chilenos_y_sus_mascotas.pdf.

[49] Buso D.S., Queiroz L.H., Silva J.E. (2013). Epidemiological aspects of dog bites considering biter dogs and victims. Veterinária Zootec..

[50] Araus D.A. (2009). “Características Demográficas, Sanitarias, de Manejo y Mordeduras Denunciadas de la Población Canina, Durante el Periodo 2008–2009, en la Ciudad de Puerto Aysén, Chile”. Director: Rafael Tamayo. Universidad Austral de Chile, Facultad de Ciencias Veterinarias, Instituto de Medicina Preventiva Veterinaria. http://cybertesis.uach.cl/tesis/uach/2009/fva663c/doc/fva663c.pdf.

[51] Pfortmueller C.A., Efeoglou A., Furrer H., Exadaktylos A.K. (2013). Dog bite injuries: Primary and secondary emergency department presentations—A retrospective cohort study. Sci. World J..

[52] Oxley J.A., Christley R., Westgarth C. (2018). Contexts and consequences of dog bite incidents. J. Vet. Behav..

[53] Schalamon J., Ainoedhofer H., Singer G., Petnehazy T., Mayr J., Kiss K., Höllwarth M.E. (2006). Analysis of dog bites in children who are younger than 17 years. Pediatrics.

[54] Kennel Club De Chile (2018). Estadísticas de inscripciones de camadas.

[55] De Keuster T., Lamoureux J., Kahn A. (2006). Epidemiology of dog bites: A Belgium experience of canine behaviour and public health concerns. Vet. J..

[56] Mills D.S., Levine E. (2006). The need for a co-ordinated scientific approach to the investigation of dog bite injuries. Vet. J..

[57] Olson K.R., Levy J.K., Norby B., Crandall M.M., Broadhurst J.E., Jacks S., Barton R.C., Zimmerman M.S. (2015). Inconsistent identification of pit bull-type dogs by shelter staff. Vet. J..

[58] Rosado B., García-Belenguer S., León M., Palacio J. (2009). A comprehensive study of dog bites in Spain, 1995–2004. Vet. J..

[59] Mohtasham-Amiri Z., Pourmarzi D., Razi M. (2015). Epidemiology of dog bite, a potential source of rabies in Guilan, north of Iran. Asian. Pac. J. Trop. Dis..

[60] Ibarra L., Espínola F., Echeverría M. (2006). Una prospección a la población de perros existente en las calles de la ciudad de Santiago, Chile. Av. Cienc. Vet..

[61] Taylor L.H., Wallace R.M., Balaram D., Lindenmayer J.M., Eckery D.C., Mutonono-Watkiss B., Nel L.H. (2017). The role of dog population management in rabies elimination—A review of current approaches and future opportunities. Front. Vet. Sci..

[62] Collinson A., Bennett M., Brennan M.L., Dean R.S., Stavisky J. (2020). Evaluating the role of surgical sterilisation in canine rabies control: A systematic review of impact and outcomes. PLoS Negl. Trop. Dis..

[63] FAO (2014). Dog population management. Report of the FAO/WSPA/IZSAM Expert Meeting–Banna, Italy, 14–19 March 2011.

[64] Pinillos R.G. (2018). One Welfare: A Framework to Improve Animal Welfare and Human Well-Being.

[65] Matthias J., Templin M., Jordan M.M., Stanek D. (2015). Cause, setting and ownership analysis of dogs bites in Bay Country, Florida from 2009 to 2010. Zoonoses Public Health.

[66] Caffrey N., Rock M., Schmidtz O., Anderson D., Parkinson M., Checkley S.L. (2019). Insights about the epidemiology of dog bites in a canadian city using a dog aggression scale and administrative data. Animals.

[67] Ghannad M.S., Roshanaei G., Rostampour F., Fallahi A. (2012). An epidemiologic study of animal bites in Ilam Province, Iran. Arch. Iran. Med..

[68] Loder R.T. (2019). The demographics of dog bites in the United States. Heliyon.

[69] Quirk J.T. (2012). Non-fatal dog bite injuries in the USA, 2005–2009. Public Health.

[70] Ósúilleabháin O. (2015). Human hospitalisations due to dog bites in Ireland (1998–2013): Implications for current breed specific legislation. Vet. J..

[71] Armstrong W., Ulloa G. (2016). Aspectos epidemiológicos sobre mordeduras caninas durante el año 2011 en la ciudad de Temuco, Chile. Sustain. Agric. Food Environ. Res..

[72] Bhuvaneswari B., Lakshmi L. (2012). Lesiones no intencionales en niños en entornos de escasos recursos: ¿hacia dónde apuntan los dedos?. Arch. Dis. Child..

[73] Hampson S.E., Andrews J.A., Barckley M. (2008). Predictores infantiles del consumo de marihuana en adolescentes: Búsqueda temprana de sensaciones, afiliación desviada de pares e imágenes sociales. Addict. Behav..

[74] Chen Y., Gao Y., Zhou L., Tan Y., Li L. (2016). A comparative study of dog-and cat-Induced injury on incidence and risk factors among children. Int. J. Environ. Res. Public Health.

[75] Schwebel D.C., Morrongiello B.A., Davis A.L., Stewart J., Bell M. (2012). The Blue Dog: Evaluation of an interactive software program to teach young children how to interact safely with dogs. J. Pediatric Psychol..

[76] Doggonesafe: Be a Tree Program. https://doggonesafe.com/FAQ_Be_a_Tree_content_issues.

[77] Gobierno Regional Metropolitano de Santiago Programa Regional Integral de Control y Prevención de la Población Canina en la Región Metropolitana de Santiago. http://www.cuidadoconelperro.cl/wp-content/uploads/2015/08/Manual-TRM-WEB.pdf.

[78] Reisner I.R., Nance M.L., Zeller J.S., Houseknecht E.M., Kassam-Adams N., Wiebe D.J. (2011). Behavioural characteristics associated with dog bites to children presenting to an urban trauma centre. Inj. Prev..

[79] Wankhede V., Waingankar P., Anjenaya S., Telang B.T. (2013). Epidemiological Study of Dog Bite Cases Reported at ARV Clinic of Rural Hospital, Panvel in Raigad District of Maharashtra, India. Int. J. Recent Trends Sci. Tech..

[80] Bustamante S. (2008). Demografía en las Poblaciones de Perros y Gatos en la Comuna de Santiago.

[81] Illanes Achondo J.J. Demografía en las Poblaciones de Perros y Gatos en el Área Rural y Urbana de la Comuna de Calera de Tango. http://repositorio.uchile.cl/handle/2250/131084.

[82] Rojas Roco A. (2005). Demografía en las poblaciones de perros y gatos en la Comuna de Lo Prado.

[83] CADEM El Chile que Viene Mascotas. https://www.cadem.cl/wp-content/uploads/2019/06/Chile-que-viene_Mayo-2019.pdf.

[84] UNDP (2003). Human Development Report 2003. Millennium Development Goals—A Compact among Nations to End Human Poverty. New York. http://hdr.undp.org/en/content/human-development-report-2003.

[85] Horwitz D., Debra F., Horwitz (2017). Blackwell’s Five-Minute Veterinary Consult Clinical Companion: Canine and Feline Behavior.

[86] Overall K. (2013). Manual of Clinical Behavioral Medicine for Dogs and Cats.

[87] Julien D.A., Sargeant J.M., Filejski C., Harper S.L. (2020). Ouch! A cross-sectional study investigating self-reported human exposure to dog bites in rural and urban households in southern Ontario, Canada. Zoonoses Public Health.

[88] Ministerio de Desarrollo Social de Chile Encuesta CASEN 2017. http://observatorio.ministeriodesarrollosocial.gob.cl/casen-multidimensional/casen/docs/Resultados_vivienda_casen_2017.pdf.

